# Hospital infection after major amputations

**DOI:** 10.1186/1476-0711-9-15

**Published:** 2010-05-19

**Authors:** José Maria Pereira de Godoy, Janalice Vasconcelos Ribeiro, Lívia Andrioli Caracanhas, Maria de Fátima Guerreiro Godoy

**Affiliations:** 1 Department of Cardiology and Cardiovascular Surgery, Medicine School of São José do Rio Preto-FAMERP and Research CNPq (National Council for Research and Development), Brazil; 2 Department of Cardiology and Cardiovascular Surgery, Medicine School of São José do Rio Preto-FAMERP, Brazil; 3 Medicine School in São José do Rio Preto (FAMERP) and Research CAPES (Coordination of Improvement of Personal of Superior Level), Brazil

## Abstract

The aim of the current study was to evaluate the prevalence of stump infections after major amputations of the lower extremities.

Patients rehospitalized in Hospital de Base of the Medicine School in São José do Rio Preto in the period from January 2005 to January 2007 due to stump infection after major amputations of lower extremities were evaluated in a retrospective study. All the patients underwent prophylactic antibiotic therapy at the time of the surgery. The Fisher exact test was utilized for statistical analysis with an alpha error of 5% (p-value < 0.05) being considered acceptable.

A total of 231 patients were submitted to major amputations during this period and 17 (7.3%) were rehospitalized due to amputation stump infections of which 5 (29.4%) died within one month. The association between death due to stump infection and other causes of death during rehospitalizations was not significant (Fisher exact test: p < 0.1). However, death during rehospitalizations was significantly higher than in the initial hospitalization.

## Introduction

Chronic critical limb ischemia, defined as > 2 weeks of rest pain, ulcers, or tissue loss attributed to arterial occlusive disease, is associated with great loss of both limb and life [1]. Major limb amputation is often required by patients with a limited capacity to tolerate post-operative complications [2]. Complications after major amputations such as wound infections, development of phantom pain, severe mental distress, myocardial infarction or strokes are frequent [2,3]. Surgeon preference, surgeon experience, physician supply, geographic influences, healthcare delivery systems, and socioeconomic factors have all been cited as influencing the treatment of patients with critical limb ischemia [4,5].

Amputation stump infection is common and may necessitate re-amputation, potentially exposing a vulnerable patient to further serious complications. Prophylactic antibiotics significantly reduced rates of stump infection in all studies, and were associated with a reduced rate of re-amputation in one [2]. The use of a prolonged 5-day course of combined antibiotics after major lower limb amputation to reduce stump infection rates also led to shorter in-hospital stays [6].

Wound infection rates ranging from 13 to 40% have been reported following major lower limb amputation [7-11]. Methicillin-resistant Staphylococcus aureus (MRSA) infection in vascular patients is associated with increased morbidity and mortality [12-14]. The aim of the current study was to evaluate the prevalence of stump infection after major amputations of lower extremities.

## Method

Hospital infection after major amputations of lower extremities performed in the period from January 2005 to January 2007 was evaluated in a retrospective study in Hospital de Base of the Medicine School in São José do Rio Preto. A total of 231 patients were evaluated in the perioperative period of major amputations of the lower extremities and the cause of death and the prevalence of hospital infection of the amputation stump were identified. All patients were submitted to prophylactic or therapeutic antibiotic treatment in the surgery; one of the following drugs was utilized at random: ciprofloxacin, cefalexin and clindamycin. For patients with leg wounds, a culture and antibiogram were performed routinely in all the open wounds and the antibiotic was adjusted as necessary. Percentages and the Fisher exact test were utilized for statistical analysis with an alpha error of 5% (p-value < 0.05) being considered acceptable.

The study was approved by the Research Ethics Committee of the Medicine School in São José do Rio Preto (FAMERP).

## Results

Of the 231 patients submitted to major amputations in this period, 17 (7.3%) suffered infections of the amputation stump, 5 (29.4%) of whom died. The overall death rate of the 231 amputees was 5.6%. No significant difference was seen between death due to stump infection and to other causes in the rehospitalizations (Fisher exact test: p < 0.1). However, the death rate after rehospitalizations was higher than in the initial hospitalization (Fisher exact test: p = 0.004). The most common infections involved were: Methicillin-resistant *Staphylococcus aureus *(MRSA) Pseudomonas aeruginosa, Enterococcus, Escherichia coli, Proteus mirabilis and Klebsiella pneumoniae.

## Discussion

The current study shows that the perioperative death of patients with hospital infection of the amputation stump was similar to death rates due to other causes. However, the small study sample size may have influenced this result. Nevertheless, other published studies have demonstrated similar overall perioperative death rates [15,16] and that infections did not have an effect on the death rate of patients [17,18]. Although infection did not interfere in the overall death rate, it was higher at rehospitalizations than in the initial hospitalization.

These patients received empirical prophylactic antibiotic therapy utilizing ciprofloxacin, cefalexin and clindamycin. For patients with leg wounds, a culture and antibiogram were performed and the treatment of infections was then reevaluated depending on the test results. All the patients were released from hospital on antibiotic therapy for periods of more than 5 days. Studies have shown that antibiotic therapy for more than 5 days is more efficacious in reducing infection rates than periods of only 24 hours [6]. These data suggest that further studies are required to determine the ideal length of antibiotic therapy after amputations. There are few studies that evaluate antibiotic therapy after amputations however the results of the publications that exist suggest that antibiotics are essential [2]. Apart from the reduction of infection, therapy reduces the necessity of re-amputations and the risks of death and morbidity of a further surgery.

The hospital costs, the emotional affect related to a further surgery, the bandaging and all the complications due to infection should be evaluated. Hence, infection is one of the main complications to be prevented and treated in these patients. The type of prophylactic antibiotics should be evaluated and treatment of established infections should be conducted using cultures and antibiograms. Other factors that patients present, such as malnutrition, may interfere in the healing of wounds and should be evaluated. Nutritional status was not part of the aim of this study and was not routinely assessed, but remarks warn about its importance. Additionally, attention should be paid to socioeconomic and cultural factors that may influence the care of these patients as the surgeon's recommendations are frequently not carried out. In this study, these aspects were identified and may have had an effect on the infection and even the death of amputees.

## Conclusion

Rehospitalization for stump infections after major amputations caused a higher mortality rate than in the initial hospitalization however it was not higher than deaths resulting from other causes of rehospitalizations.

## Competing interests

The authors declare that they have no competing interests (political, personal, religious, ideological, academic, intellectual, commercial or any other) in relation to this manuscript.

## Authors' contributions

JMPG, JVR, LAC and MFGG participated and contributed to all phases of the study. All authors read and approved the final manuscript.
